# Microbiota, Prostatitis, and Fertility: Bacterial Diversity as a Possible Health Ally

**DOI:** 10.1155/2021/1007366

**Published:** 2021-09-28

**Authors:** Jenniffer Puerta Suárez, Walter D. Cardona Maya

**Affiliations:** Reproduction Group, Department of Microbiology and Parasitology, School of Medicine, University of Antioquia-UdeA, Medellin, Colombia

## Abstract

**Background:**

In health, microorganisms have been associated with the disease, although the current knowledge shows that the microbiota present in various anatomical sites is associated with multiple benefits.

**Objective:**

This study aimed to evaluate and compare the genitourinary microbiota of chronic prostatitis symptoms patients and fertile men.

**Materials and Methods:**

In this preliminary study, ten volunteers have included 5 volunteers with symptoms of chronic prostatitis (prostatitis group) and five fertile volunteers, asymptomatic for urogenital infections (control group) matched by age. Bacterial diversity analysis was performed using the 16S molecular marker to compare the microbiota present in urine and semen samples from chronic prostatitis symptoms and fertile volunteers. Seminal quality, nitric oxide levels, and seminal and serum concentration of proinflammatory cytokines were quantified.

**Results:**

Fertile men present a greater variety of operational taxonomical units-OTUs in semen (67.5%) and urine (17.6%) samples than chronic prostatitis symptoms men. Chronic prostatitis symptoms men presented a higher concentration of IL-12p70 in seminal plasma. No statistically significant differences were observed in conventional and functional seminal parameters. The species diversity in semen samples was similar in healthy men than prostatitis patients, inverted Simpson index median 5.3 (5.0–10.7) vs. 4.5 (2.1–7.8, *p*=0.1508). Nevertheless, the microbiota present in the semen and urine samples of fertile men presents more OTUs. Less microbial diversity could be associated with chronic prostatitis symptoms. The presence of bacteria in the genitourinary tract is not always associated with the disease. Understanding the factors that affect the microbiota can implement lifestyle habits that prevent chronic prostatitis.

**Conclusion:**

Chronic prostatitis does not seem to affect male fertility; however, studies with a larger sample size are required. Our preliminary results strengthen the potential role; the greater bacterial diversity is a protective factor for chronic prostatitis.

## 1. Introduction

Chronic prostatitis is a frequent and multicausal condition. Among the causes of prostatitis that include infections [1], mainly bacterial, however, identifying the causative agent is not always possible, and in addition, the gland can harbour microbiota. With sequencing techniques, it is possible to identify a great variety of microbiota, its effects on health, and its effect on the immune system [2]. In chronic bacterial prostatitis men, an improvement in symptoms, life quality, and less antibiotic consumption have been described with the oral administration of *Lactobacillus casei* [3], suggesting the close relationship between gastrointestinal and urogenital microbiota [4].

Prostatitis is the most common urological diagnosis in young men under 50 years after benign prostatic hyperplasia and prostate cancer [5]. The primary causes of the illness such as infections, immunologic status, urine reflux, and mental stress have been identified as primary causes [1]. Clinically, prostatitis is classified into four types: (i) acute bacterial prostatitis, (ii) chronic bacterial prostatitis, (iii) chronic pelvic pain syndrome, and (iv) asymptomatic inflammatory prostatitis [6–8]. Chronic bacterial prostatitis is responsible for 5–10% of total prostatitis cases, of which at least 30% is associated with recurrent urinary infections [8]. Up to 90% of patients are classified as having chronic pelvic pain syndrome because the cause cannot be identified. The gold standard for prostatitis is the four-vessel test [9]; however, at present, it is not performed routinely due to the risk of causing bacteremia [7].

Furthermore, this test only detects those culturable microorganisms, so new techniques are necessary to identify all the organisms in the gland responsible for the disease. The use of next-generation sequencing (NGS) techniques could improve the understanding of the microbiome [10], especially dysbiosis caused by prostatitis and its impact on health. Bacterial diversity analysis (metataxonomics) uses the 16S molecular marker (variable regions V3–V4) to assess men's microbiota. Besides, the semen sample has higher sensitivity than expressed prostatic excretion (EPS) for diagnosing chronic bacterial prostatitis [11], and it is also practical and comfortable for the patient. Therefore, this preliminary study aimed to determine men's microbiota with prostatitis-like symptoms and their impact on seminal quality.

## 2. Materials and Methods

### 2.1. Study Participants

The protocol and informed consent form were approved by the Bioethics Committee for Research in Humans at the Institute of Medical Research, School of Medicine, University of Antioquia (act number 006), in April 2018. All patients provided written informed consent regarding their participation and publication of their clinical data. Five chronic prostatitis symptoms and five fertile men asymptomatic for urogenital infections volunteers were included. All men were generally in good health, without sexually transmitted diseases. None was under antibiotic treatment at the time of the sampling. National Institute of Health of Chronic Prostatitis Symptoms Index (NIH–CPSI [12]) translated and validated into Spanish [13] was employed to select the volunteers according to the criteria reported by Nickel et al., [5].

Each volunteer gave a semen sample and a midstream urine sample; both samples replaced the prostate fluid sample obtained through stimulation of the gland through the rectum [14]. In addition, a blood sample was taken by qualified personnel in a nonanticoagulated vacutainer tube (Becton Dickinson, NJ, USA) to obtain the serum. The donors also filled out a survey with sociodemographic factors, lifestyle, urinary symptoms, and other relevant aspects of sexual and reproductive health to identify factors associated with prostatitis symptoms.

### 2.2. Semen and Seminal Parameters

Prior to semen analysis, all donors were asked to abstain from sexual intercourse or masturbation for 3–5 days before semen collection and delivered to the laboratory within 1 hour of ejaculation.

### 2.3. Conventional Seminal Parameters

After semen liquefaction was completed, each semen sample was analyzed for conventional parameters: sperm motility, vitality, concentration, total sperm concentration, and sperm morphology according to those established by the World Health Organization in the fifth edition of its Human Semen Processing Manual, while the sperm concentration was evaluated using the Makler chamber [15, 16].

### 2.4. Functional Seminal Parameters

Sperm mitochondrial membrane potential [17], sperm membrane integrity [18], chromatin structure assay [19], sperm membrane lipoperoxidation [20], and intracellular levels of reactive oxygen species (ROS) [17] were evaluated by flow cytometry (Fortessa, Becton Dickinson, NJ, USA), according to previously established protocols, analyzing between 5,000 and 10,000 sperm cells. Data analysis was carried out with FlowJo 7.6 (TreeStar Inc., Oregon, USA).

### 2.5. Seminal Plasma Total Antioxidant Capacity

For this test, 3 mL of DPPH (2,2-diphenyl-1-picrylhydrazyl) was mixed with 200 *μ*L of the sample. After one hour of incubation, the sample was read in a spectrophotometer (Spectronic 20 spectrophotometer®, Genesys, Rochester, NY, USA) at 515 nm, used ascorbic acid as a positive control [21].

### 2.6. Prostate-Specific Antigen (PSA)

According to the manufacturer's instructions, PSA was quantified using the commercial total PSA kit (DiaMetra, Perugia, Italy). Prostate antigen values greater than 4 ng/mL were considered positive.

### 2.7. Nitric Oxide Quantification

Nitrite quantification was performed using the commercial Griess reagent kit for nitrite determination (Molecular probes, Oregon, USA) according to the manufacturer's instructions and after deproteinization of the semen and serum samples according to the Serafini method [22].

### 2.8. Cytokine Quantification

Quantification of the cytokines IL-12p70, IL-10, IL-1*β*, IL-6, IL-8, TNF, IL-2, IL-4, IL-10, IL-17, and INF-*γ* was performed using the kits BD Cytometric Bead Array (CBA) Human Inflammatory Cytokines and Human Th1/Th2/Th17 Cytokine Kit (Becton Dickinson, NJ, USA). The analysis was carried out in the FlowJo 7.6.

### 2.9. Bacterial Diversity Analysis

DNA extraction was performed using the Stool DNA Isolation Kit (Norgen) to identify the microbiota, and gDNA quantification was performed by the PicoGreen colourimetric method. Subsequently, control of 16S gene amplification was performed using the primers 27F (AGAGTTTGATCCTGGCTCAG) and 1492R (TACGGYTACCTTGTTACGACTT), and a 1500 base pair (bp) fragment was successfully amplified for all samples. For sequencing, the 16S ribosomal gene variables V3 and V4, the Bakt_341F (CCTACGGGNGGCWGCAG), and Bakt_805R (GACTACHVGGGTATCTAATCC) oligonucleotides were used. Sequencing was performed on the platform MiSeq Illumina, generating paired reads 300 bases each. Sequence quality analysis and classification were developed in MetaCoMET (Metagenomics Core Microbiome Exploration Tool, MetaCoMET).

### 2.10. Statistical Analysis

A chi-square and a Mann–Whitney test were used to compare the dichotomous and numerical variables between both groups. The data were analyzed using the statistical program GraphPad Prism 6.0 (GraphPad, San Diego, CA, USA), and a value of *p* < 0.05 was considered significant.

## 3. Results

The median and range of age, abstinence period, and body mass index of the fertile group and prostatitis-like symptoms patients included in the study was 34 (21–44) years and 34 (21–44) years (*p* > 0.999), 2 (2–5) and 3 (2–5) days (*p*=0.9206), and 26.4 (24.4–30.9) and 22.9 (19.7–26.8) kg/m^2^ (*p*=0.0952), respectively.

Semen and urine microbiota of five fertile volunteers and five chronic prostatitis symptoms volunteers matched by age, and classified using the NIH–CPSI were compared (Table 1).

When comparing the conventional and functional semen analyses of prostatitis-like symptoms men and fertile men matched by age, we did not find statistically significant differences (Table 2). All conventional seminal parameters were in both groups above the lower limit of reference according to the WHO [23]. In addition, no PSA serum concentration differences were observed (Table 2). The median level of IL-12p70 in seminal plasma was significantly increased in chronic prostatitis symptoms volunteers compared to the fertile group. Also, there was no difference in other cytokine concentrations between both groups (Table 3).

**Figure 1:**
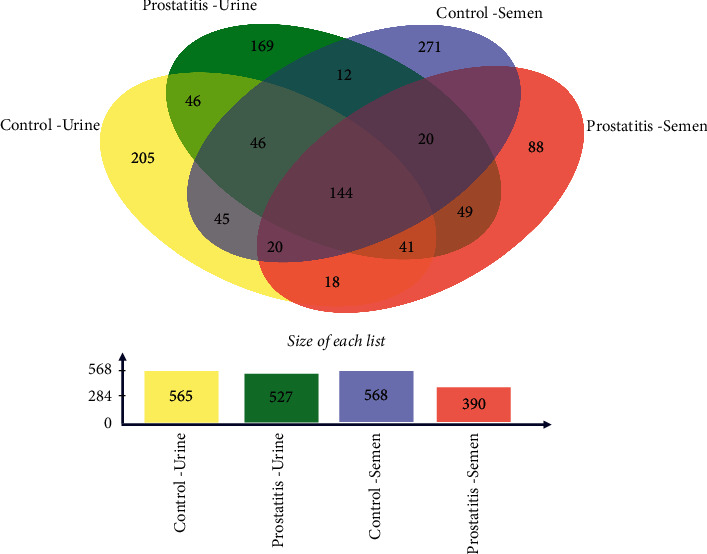
Venn diagram of OTU overlapping in urine and semen samples. (a) Venn diagram obtained with semen and urine samples from chronic prostatitis symptoms and fertile men showing that both types of samples in both groups share 144 OTUs. (b) Semen and urine samples from volunteers with chronic prostatitis showing a lower amount of OTU than samples from fertile men in the control group.

**Figure 2:**
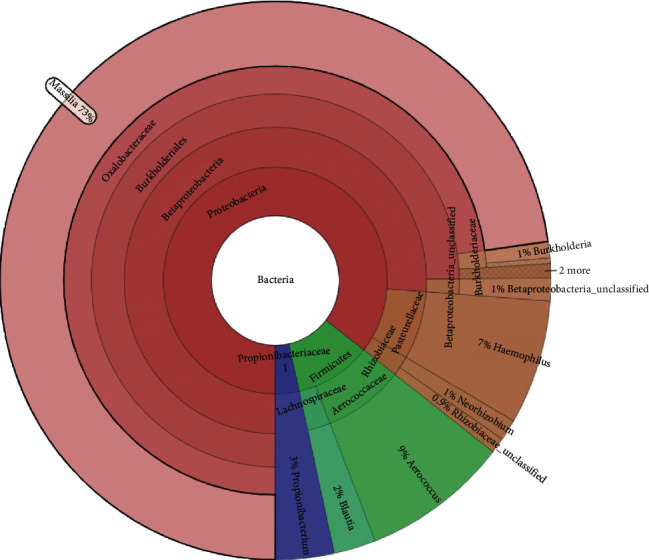
Different OTUs between prostatitis-like symptoms men and fertile men. Semen and urine samples from chronic prostatitis symptoms and fertile men differ statistically in 14 OTU. Most of them are not detectable by traditional culture techniques, which show the importance of sequencing in the study of prostate disease.

When evaluating the genitourinary microbiota, it is observed that the species diversity in semen samples was similar in healthy men than prostatitis patients, inverted Simpson index median 5.3 (5.0–10.7) vs. 4.5 (2.1–7.8, *p*=0.1508). Nevertheless, the microbiota present in the semen and urine samples of fertile men presents 67.5% and 17.6% more OTUs, respectively, than prostatitis-like symptoms volunteer (Figure 1). We also observe men with prostatitis and fertile men share 144 operational taxonomic units (OTU). We also found no differences in the urine sample (inverted Simpson index median control 6.2 (4.5–6.8) vs. prostatitis 4.8 (4.3–12.2), *p*=0.8016). Finally, we observed statistically significant differences in 14 OTUs in the different samples of the groups (Figure 2).

## 4. Discussion

This preliminary study evaluated some factors associated with chronic prostatitis by comparing fertile men with no urogenital infections and men with chronic prostatitis in a small sample of volunteers. We explored the microbial content of the semen and urine of men to evaluate the prostatitis effect on seminal parameters and fertility. We compared the conventional and functional seminal parameters of prostatitis symptoms with men who had a pregnant partner or children under two years of age.

We observed no differences in semen quality between both groups. In fact, the seminal parameters of the volunteers in both groups were higher than the WHO lower reference limit. Additionally, men with prostatitis also presented higher seminal concentration and high concentrations of IL-12p70 in serum. This proinflammatory cytokine is secreted mainly by macrophages and monocytes, stimulating the production of IFN-*γ*, which suggests a predominance of Th1 lymphocyte activation, facilitating the establishment of an inflammatory environment that becomes chronic [24].

The microbiome is composed of genetic material and microorganisms. It is also considered more complex than the human genome, and the microbiota refers to the population of bacteria present in various anatomical sites [10]. Although prostatitis is a multicausal condition, genitourinary infections are included among its causes, and the majority of bacterial prostatitis follows a urinary tract infection [1, 6]. However, the presence of microorganisms does not always imply disease, and caution is required in interpreting the microbiological results of urinary tract samples until the microbiota of this anatomical site is correctly established. An adequate association between symptoms and the detection of microorganisms should contribute to the diagnosis of prostatitis.

Bacterial diversity is a crucial factor in preventing the appearance of genitourinary diseases. The urine of prostatitis-like symptoms men presented 17.6% less diversity of OTUs than fertile men. This result was higher in the semen sample since 67.5% fewer OTUs were observed in the semen of prostatitis-like symptoms men. The urogenital tract microbiota of men with and without symptoms of prostatitis includes bacteria *Rhizobiaceae, Burkholderia, Achromobacter, Delftia, Campylobacter, Ezakiella, Anaerococcus, Prevotella, Haemophilus*, and *Porphyromonas.* Specifically, in urine, the most common bacterial genera in men with and without symptoms of prostatitis are *Pantoea, Geobacillus, Kocuria, Veillonella, Brevibacterium, Pseudomonas, Acetobacteraceae, Neisseria, Chryseobacterium,* and *Dialister* and in semen are *Weissella, Proteobacteria, Burkholderiales, Achromobacter, Campylobacter*, and *Prevotella* because today's available technique makes that some microorganisms are uncultivable in traditional microbial evaluation methods that are obsolete. *Prevotella* appeared to exert a negative effect on sperm quality [10]. *Firmicutes* (especially *Lactobacilli*), *Bacteroidetes, Proteobacteria*, and *Actinobacteria* comprise the highest proportion of the seminal microbiome [25].

In this preliminary study, we only found statistically significant differences in the presence of fourteen OTUs. These fourteen OTUs explain the difference in the microbiota of prostatitis-like symptoms and fertile men: *Burkholderiaceae, Achromobacter, Aerococcus, Blautia, Burkholderiales, Propionibacterium, Betaproteobacteria, Haemophilus, Burkholderia, Massilia, Rhizobiaceae*, and *Neorhizobium*. In general, they are little-known microorganisms in the clinical field, so sequencing is a powerful tool that allows us to discover the world surrounding enigmatic infectious diseases such as prostatitis [26–28]. Culture-based studies detected fewer anaerobic bacteria than NGS [10]. Few are the cases described for prostatitis caused by the *Burkholderia* genus. However, pulmonary infections by this microorganism that spread through the hematogenous route can reach the prostate causing chronic prostatitis [29]. The particularities in the microbial culture have made the accurate diagnosis of genitourinary infections difficult, as in *Corynebacterium urealyticum*, a Gram-positive, facultative anaerobic bacillus that is difficult to grow, responsible for prostatitis that is also associated with calcifications in the prostate [30]. Molecular tools allow the diagnosis of hidden infections in the light of traditional microbiological culture techniques.

Nevertheless, sequencing costs today are higher than traditional bacteriological methods; the microorganism's effect on fertility is still under discussion. Much more research is required to establish the microbiota of health and disease and validate powerful tools such as sequencing for daily use in the clinic. Microbiota studies are novel and have shown bacteria as a protective factor against disease; it should not be forgotten that bacteria can negatively impact sperm function [10]. However, studies evaluating the microbiota have used semen or urine samples, which could be biased by contaminating the sample collection [31].

Prostatitis is associated with affected male fertility, during illness can be affected semen quality, especially sperm concentration, motility, vitality, and morphology [25]. Semen and vaginal discharge are not sterile. The bacterial microbiome impacts on fertility and pregnancy [10], and this microbiome can be changed all the time, depending on such environmental factors and interaction with other organisms [31].

In addition to aerobic bacteria, anaerobes can also cause chronic bacterial prostatitis, mainly due to microorganisms such as *Peptostreptococcus* spp. and *Bacteroides* spp. Knowledge about these infections is limited by the diagnostic methods, causing underestimating of the natural role of anaerobes [6].

The main limitations that affected the study quality included selecting participants using the NIH–CPSI without diagnostic imaging techniques, digital rectal examination, the use of the four-vessel test, and the limited number of participants in both groups. The selection of participants and the similarities of the patients, primarily in terms of age and most of the evaluated characteristics, allow us to somehow rule out the impact of lifestyle habits, features, and sexual behaviours over genitourinary microbiota.

## 5. Conclusion

Chronic prostatitis does not seem to affect seminal quality; however, more studies are required. The greater bacterial diversity of the genitourinary microbiota could be a protective factor for chronic prostatitis in men. Studying the factors associated with this greater microbial diversity will allow the establishment of healthy behaviours that limit the appearance of genitourinary diseases in men.

## Figures and Tables

**Table 1:** NIH–CPSI classification of volunteers.

Domain (score)	Symptoms classification, median (range)		*P* value
	Fertile group, control	Prostatitis-like symptoms	
Pain (0–21)	0 (0-1)	11 (7–11)	0.0079
Urinary symptoms (0–10)	1 (0–3)	7 (6–10)	0.0079
Quality of life impact (0–12)	0 (0–2)	4 (2–5)	0.0159
Total score (0–43)	1 (0–5)	20 (20–26)	0.0079

**Table 2:** Seminal quality and oxidative stress in prostatitis-like symptoms men and fertile men.

	Fertile group, control, median (range)	Prostatitis-like symptoms, median (range)	*P* value
Seminal volume (mL)	4 (1.5–4.5)	3 (1.5–7.5)	0.7460
Progressive motility (%)	55.5 (41.5–81.0)	57 (6.0–64.0)	0.6667
Nonprogressive motility (%)	6.5 (2.0–16.0)	6.0 (1.0–25.0)	0.9524
Immotile spermatozoa (%)	39.0 (17.0–45.0)	42.0 (29.0–90.0)	0.8016
Concentration/mL (10^6^/mL)	80.0 (40.5–270.0)	205.0 (7.0–254.0)	0.9444
Total concentration (10^6^/ejaculate)	178.2 (115.5–1080.0)	431.8 (22.4–1538.0)	0.8016
Viability (%)	79.0 (77.0–90.0)	70.0 (49.0–85.0)	0.1667
Sperm with normal morphology (%)	4.8 (4.2–8.6)	4.4 (2.8–6.8)	0.8016
Teratozoospermia index	1.4 (1.1–1.5)	1.2 (1.1–1.5)	0.2222
High mitochondrial membrane potential (%)	63.2 (44.2–7.5)	50.0 (12.3–55.5)	0.0556
Plasma membrane integrity (%)	64.8 (48.1–84.4)	35.8 (6.2–69.7)	0.1508
ROS production (%)	64.3 (50.5–86.2)	55.7 (17.7–61.9)	0.0556
DNA fragmentation index (%)	10.6 (10.4–11.4)	11.1 (10.5–14.3)	0.3889
Membrane lipid peroxidation (%)	49.8 (9.1–80.5)	67.0 (44.9–93.3)	0.4127
Antioxidant capacity of seminal plasma %)	61.0 (45.3–81.4)	62.0 (9.5–69.3)	0.5317
Serum nitric oxide (µM)	3.7 (2.1–13.0)	3.0 (1.7–4.1)	0.5000
Plasma nitric oxide (µM)	1.5 (0.6–1.7)	0.5 (0.2–1.9)	0.3016
PSA (ng/mL)	0.0 (0.0–1.4)	0.9 (0.0–120.0)	0.3651

Mann–Whitney test. Data indicate median and range.

**Table 3:** Concentrations of seminal cytokines in seminal and serum samples of the control group and prostatitis-like symptoms.

	Cytokine (pg/mL)	IL-12p70	IL-10	IL-1*β*	IL-6	IL-8	TNF	IL-2	IL-4	IL-10	IL-17	IFN-*γ*
Seminal samples	Fertile group	0^a^ (0–1.9)	0 (0–0.8)	0 (0–4.5)	2.2 (0–32.7)	1812.0 (997.9–3299)	0 (0–7.1)	0 (0–27.09)	0 (0–6.3)	1.5 (0.0–18.3)	5.9 (5.3–10.2)	ND
	Prostatitis-like symptoms group	45.5 (0–252.8)	1.0 (0–29.4)	1.6 (0–31.6)	6.0 (01–100.9)	1230.0 (684.1–2533)	47.75 (0–127.5)	0.0 (0.0–116.3)	0 (0–20.25)	0.9 (0.0–21.3)	8.1 (5.5–48.1)	0 (0–22.1)

Serum samples	Fertile group	0 (0–210.3)	0 (0–199.1)	0 (0–455.7)	0 (0–304.2)	24.4 (0–313.5)	0 (0–50.8)	1.0 (0.0–176.1)	2.0 (0–13.5)	0.0 (0.0–7.3)	10.4 (9.1–24.8)	0 (0–5.3)
	Prostatitis-like symptoms group	ND	ND	ND	ND	2.2 (0.0–11.5)	0.0 (0.0–1.4)	0.0 (0.0–21.9)	4.5 (0–10.5)	0 (0–3.1)	17.1 (11.2–53.7)	0 (0–27.3)

Data indicate median (range). ND, not detected. ^a^Significantly different from the control group: *p* < 0.05 (Mann–Whitney *U* test).

## Data Availability

The database used to support the findings of this study is available from the corresponding author upon request.
